# High-Precision Time Synchronization and Autonomous Maintenance for LEO Satellite Constellations Based on High-Stability Crystal Oscillators

**DOI:** 10.3390/s26061839

**Published:** 2026-03-14

**Authors:** Lei Mu, Xiaogong Hu, Mengjie Wu, Jin Li

**Affiliations:** 1 Shanghai Astronomical Observatory, Chinese Academy of Sciences, Shanghai 200030, China; mulei@shao.ac.cn (L.M.);; 2 School of Astronomy and Space Science, University of Chinese Academy of Sciences, Beijing 100049, China

**Keywords:** LEO satellite time synchronization, temperature sensor compensation, autonomous timekeeping, Kalman filtering, frequency drift prediction

## Abstract

In recent years, the large-scale deployment of Low Earth Orbit (LEO) constellations has made autonomous time synchronization and reference maintenance within constellations a critical enabling technology. Achieving high-precision synchronization with low cost and low power consumption, without relying on onboard atomic clocks or Global Navigation Satellite System (GNSS) signals, remains a significant challenge. This paper proposes an autonomous time synchronization method for LEO constellations that relies solely on high-stability crystal oscillators as local oscillators. By leveraging satellite-to-ground and inter-satellite measurement links, the proposed approach enables constellation-wide time synchronization without external timing references. A satellite-to-ground link visibility time model is established based on orbital parameters and ground station visibility geometry. On this basis, a discrete state-space model is constructed, incorporating temperature-induced frequency perturbation compensation, frequency offset estimation, and control voltage regulation. A combined Kalman filtering and Linear Quadratic Regulator (LQR) control framework is employed to achieve precise time offset synchronization and long-term maintenance. Experimental results demonstrate that, under a Walker-Delta constellation configuration with an orbital altitude of 800 km and an inclination of 55°, the proposed method introduces a time synchronization performance better than 5 ns (1σ), with a peak-to-peak error below 30 ns. This level of performance satisfies the timing requirements of typical LEO constellation applications, including communication scheduling, high-rate modulation, and critical infrastructure timing services. Moreover, the proposed scheme supports decentralized deployment and provides local physical time signal outputs, making it well suited for large-scale satellite networks requiring high-precision autonomous time synchronization.

## 1. Introduction

### 1.1. Research Background and Significance

Low Earth Orbit (LEO) satellites, characterized by relatively low manufacturing and launch costs as well as short communication latency, are well suited for large-scale deployment and have become a key development direction for future constellation-based navigation, communication, and remote sensing systems. In recent years, dozens of organizations worldwide have announced plans for LEO satellite constellations. It is expected that the total number of operational LEO satellites will exceed 50,000 upon completion, representing several tens of times the number of satellites currently in orbit. Representative projects include SpaceX’s Starlink constellation and the Iridium NEXT system abroad, as well as the Hongyan constellation proposed by the China Aerospace Science and Industry Corporation and the “GW” constellation planned by China Electronics Technology Group.

In LEO satellite systems, precise time synchronization is essential for ensuring communication stability, positioning accuracy, and coordinated operation among network nodes [1]. In particular, broadband LEO satellite communication scenarios, such as link access, beam switching, and TDMA resource scheduling, rely on synchronization accuracy at the nanosecond level. Existing studies indicate that improved Timing Advance methods can constrain link alignment errors to within tens of microseconds in LEO communication networks, while further enhancement to nanosecond-level synchronization can significantly improve system throughput and spectral efficiency [2]. In inter-satellite communication scenarios employing high-frequency modulation schemes such as OFDM, time synchronization errors must be jointly modeled with carrier frequency offsets and constrained to the nanosecond level; otherwise, signal demodulation performance will be severely degraded [3].

Therefore, a time synchronization accuracy on the order of 100ns represents a fundamental performance threshold for typical LEO constellation communication scenarios, including link access, beam switching, and high-frequency modulation. Under this background, achieving high-precision, low-cost, and low-power time synchronization and reference maintenance within a constellation has become a key enabling technology for distributed satellite collaboration. At present, most LEO satellites are not equipped with high-precision onboard time-frequency facilities and primarily rely on GNSS receivers for time synchronization [4]. However, this approach suffers from significant limitations in weak or GNSS-denied environments caused by signal blockage, interference, or hostile conditions. Consequently, investigating autonomous timekeeping and time maintenance under minimal external dependency is of substantial theoretical and engineering significance.

### 1.2. Related Work

On the domestic side, the BeiDou-3 navigation system is equipped with Ka-band inter-satellite links and high-precision rubidium clocks, enabling star–ground synchronization and joint timekeeping across the entire constellation based on master ground stations [5,6,7]. Several research institutions have also explored techniques such as remote control of onboard crystal oscillators and common-view star–ground synchronization [8,9,10]. Nevertheless, research efforts focusing on low-cost, lightweight, and autonomous timekeeping solutions for LEO constellations remain at an early stage [11,12].

Internationally, NASA proposed a unified space–time architecture centered on GPS in its Space Communications and Navigation Architecture Recommendations (2005–2030), aiming to achieve autonomous navigation and timekeeping through inter-satellite links and onboard atomic clocks [13]. The European Galileo system has conducted extensive research on onboard clock management, inter-satellite measurement accuracy, and time dissemination mechanisms [14]. Projects such as ACES and PRACS have further explored the feasibility of cold atom clocks and microwave links on space platforms [15].

In recent years, standardization efforts in positioning, navigation, and timing (PNT) systems have accelerated. The IEEE P1952 standard, entitled Standard for Resilient PNT User Equipment, aims to define performance requirements for next-generation time synchronization systems operating in complex environments [16]. Its technical specifications reference the practical performance of several advanced systems, among which the synchronization performance of the Satelles Time and Location (STL) system is frequently cited. At the joint Workshop on Synchronization and Timing Systems (WSTS) and the Precise Time and Time Interval Systems and Applications Meeting (ION PTTI) held in the United States in 2023, it was reported that the STL system can achieve a typical 1σ time synchronization performance of 10–50ns [17,18].

### 1.3. Challenges and Open Issues

Most existing high-precision time synchronization solutions rely heavily on GNSS systems or inter-satellite links, resulting in strong dependence on spaceborne resources and supporting infrastructure. In the event of GNSS signal loss, frequency offsets and aging effects of the local oscillator can cause the internal time offset of the system to grow rapidly over time, quickly exceeding the tolerance limits required by mission operations [19]. Although onboard atomic clocks can mitigate this issue, their large size, high power consumption, and high cost make them unsuitable for large-scale constellation deployment [20]. In recent years, chip-scale atomic clocks (CSACs) have emerged as a promising alternative. For example, the SA.45s, the newest member of Microsemi’s CSAC product family, provides compact size, low power consumption (below 120mW), and excellent short-term stability on the order of $3\times 10^{-10}$ at τ=1s, together with built-in 1PPS interfaces for synchronization and holdover applications. However, compared with high-quality quartz oscillators, CSAC devices typically exhibit inferior short-term stability and phase noise performance. In addition, practical satellite timing systems still require supporting electronics for signal generation, monitoring, and control, which increases the overall system complexity and power consumption. Furthermore, quartz oscillators have been extensively studied for decades, and their temperature characteristics, aging behavior, and frequency prediction models are well established both theoretically and experimentally. Therefore, in this work, a high-stability OCXO is adopted as the local oscillator.

High-stability crystal oscillators, such as oven-controlled crystal oscillators (OCXOs), offer advantages in terms of compact size, low power consumption, and cost, typically amounting to less than 1% of that of spaceborne atomic clocks. However, their major limitation lies in poor long-term stability. Under free-running conditions, time errors can exceed system thresholds within minutes, rendering them inadequate for long-term synchronization requirements in satellite constellations [21]. Moreover, LEO satellites operate at low altitudes with high angular velocities, resulting in short star–ground visibility durations and short orbital periods. Under such constraints, when only limited synchronization resources are available, such as short-duration star–ground synchronization opportunities, compensating for the long-term instability of high-stability crystal oscillators while achieving both accuracy and stability becomes a critical challenge. Developing a lightweight and high-precision constellation time synchronization solution capable of achieving time synchronization accuracy at the 10–50ns level remains a key research problem.

### 1.4. Contributions and Technical Approach

To address the problem of time maintenance for LEO satellite constellations operating in weak GNSS environments, this paper designs a time synchronization and maintenance system based on high-stability crystal oscillators (OCXOs) combined with short-duration star–ground and inter-satellite synchronization mechanisms. The proposed system aims to provide a low-cost, low-power, and highly reliable distributed time reference solution. To accommodate different orbital altitudes and support inter-satellite synchronization, a synchronization strategy with a period of a+b minutes is designed, adopting a structure of “*a* minutes of closed-loop synchronization followed by *b* minutes of open-loop timekeeping”. During the closed-loop phase, short-duration synchronization via star–ground and inter-satellite links is employed, while during the open-loop phase, local time reference maintenance is achieved through temperature compensation and predictive control.

To evaluate the feasibility of the proposed strategy under representative constellation conditions, a Walker-Delta constellation model is adopted for simulation, with an orbital altitude of 800 km, an inclination of 55°, and a total of 36 satellites deployed to cover major regions of China. Based on orbital geometry and ground station visibility analysis, the single-pass visibility duration of the master ground station is estimated to be approximately 7 min, with an orbital period of about 100 min, providing a basis for subsequent periodic synchronization and timekeeping experiments.

In terms of control system design, a discrete-time state-space model centered on the OCXO frequency is established, incorporating key factors such as temperature disturbances, control voltage inputs, and frequency drift. During the synchronization phase, time offset measurements obtained via star–ground and inter-satellite links are combined with Kalman filtering for state estimation, and an LQR controller is employed to generate optimal control voltages for rapid correction of frequency offsets and drift trends. During phases without time offset measurements, a semi-open-loop control scheme is constructed by integrating temperature measurements and predictive compensation functions to maintain frequency stability and time error convergence.

At the current stage, due to the lack of access to real star–ground and inter-satellite time offset data, a ground-based experimental platform is employed for validation. A GNSS receiver providing a 1PPS signal is used as an equivalent master clock input, and a time interval error (TIE) measurement device is utilized to measure the time offset between the local system and the master clock, thereby validating the proposed control scheme.

Compared with traditional systems that rely on ground-based backend processing and time offset prediction, the proposed control scheme enables active frequency disciplining and dynamic compensation of the crystal oscillator, allowing the onboard system to directly output physical time signals with complete local time maintenance capability. The proposed method maintains nanosecond-level time offset control accuracy even during extended open-loop operation, significantly reducing dependence on ground information systems and facilitating the evolution of constellation systems toward autonomous and distributed architectures.

### 1.5. Innovations of This Work

- By combining typical constellation orbital geometry analysis with visibility arc distribution characteristics, a “short-duration synchronization plus long-duration timekeeping” strategy is proposed, enabling periodic time calibration and intermittent autonomous time maintenance.
- A low-GNSS-dependence time synchronization and maintenance architecture for LEO constellations is proposed, integrating closed-loop control with autonomous timekeeping.
- A discrete state-space modeling framework incorporating precise temperature disturbance compensation, frequency offset modeling, and control voltage regulation is explored in conjunction with Kalman filtering and LQR control, investigating the feasibility of achieving time maintenance accuracy better than 50ns, as specified in the IEEE P1952 standard, using only low-cost domestic crystal oscillators as onboard local oscillators.
- The proposed system directly provides a usable 1PPS physical time signal without relying on time offset estimation from an information processing system, enabling decentralized time synchronization architectures.

## 2. Materials and Methods

### 2.1. Constellation Design and Time Offset Measurement

#### 2.1.1. Constellation Parameters

A Walker constellation was adopted to emulate a representative LEO communication scenario [22]. The orbital altitude was set to 800 km, with the primary service region over mainland China. The configuration is summarized as follows:

- Constellation type: Walker Delta
- Total number of satellites *t*: 36
- Number of orbital planes *p*: 6
- Number of satellites per plane *f*: 6
- Phasing factor *F*: 6
- Inclination: approximately 55° to cover most regions of China, including high-latitude and low-latitude areas
- Orbital altitude: 800 km for all satellites
- Orbital period: approximately 100 min at 800 km altitude
- Frequency band: selected according to mission requirements, e.g., L-band for mobile satellite service or Ku/Ka-band for broadband communications
- Ground station deployment: determined by geographic location, terrain constraints, and station construction cost, with emphasis on maximizing visible arcs within the service region

#### 2.1.2. Estimation of Visibility Arc and Contact Time

Given the Earth radius, orbital altitude, and the geometry between a ground station (assumed in Beijing) and an LEO satellite, the visibility arc length *L* and contact time *t* can be estimated by:

(1) $$\left\{\begin{array}{cc} \alpha & =\arccos\left(\frac{R}{R+h}\right),\\ L & =(R+h)\cdot \alpha ,\\ v & =\sqrt{\frac{GM}{R+h}},\\ t & =\frac{L}{v}, \end{array}\right.$$

where R=6371km is the Earth radius, h=800km is the orbital altitude, $G=6.67430\times 10^{-11}\;m^{3}\;{\mathrm{kg}}^{-1}\;s^{-2}$ is the gravitational constant, and $M=5.972\times 10^{24}\;\mathrm{kg}$ is the Earth mass.

By substituting these values, we obtain

$$\alpha \approx 0.477\;\mathrm{rad},\;L\approx 3419.6\;\mathrm{km},\;v\approx 7.45\;\mathrm{km}\;s^{-1},\;t\approx 7.64\;\min.$$

Therefore, within one orbital period, the designed constellation yields a star–ground visibility window of approximately 7.5 min for a given pass over the ground station, while the remaining time requires autonomous holdover [23].

#### 2.1.3. Daily Visibility Pattern from a Single Satellite

**Figure 1:**
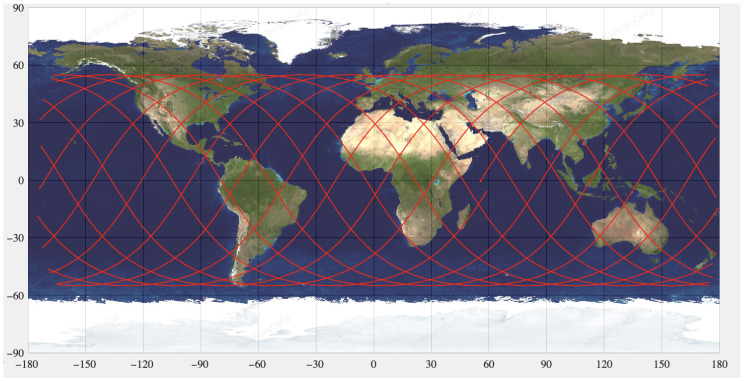
Subsatellite ground-track distribution of the considered LEO constellation.

In addition to the single-pass contact duration estimated above, it is necessary to analyze how frequently a given satellite becomes visible to the ground station during a day. The subsatellite ground-track distribution of the considered constellation is illustrated in Figure 1.

For the selected orbital altitude of 800 km, the orbital period is approximately 100–101 min, corresponding to roughly

$$\frac{24\times 60}{101}\approx 14$$

orbital revolutions per day. Due to the combined effect of orbital motion and Earth rotation, the subsatellite ground track exhibits an approximate repeat period of about 23.5 h.

Consequently, a ground station does not observe the satellite during every orbital revolution. Instead, the visibility occurs in clusters of consecutive passes. Based on the simulated results for a Beijing ground station, the typical daily visibility pattern of a single satellite can be summarized in Table 1.

Therefore, although a satellite completes about fourteen orbital revolutions per day, only about five passes provide direct satellite–ground contact. During the remaining orbital cycles without visibility, synchronization updates can be obtained through brief inter-satellite link (ISL) contacts.

#### 2.1.4. Inter-Plane Visibility in the Multi-Plane Constellation

**Figure 2:**
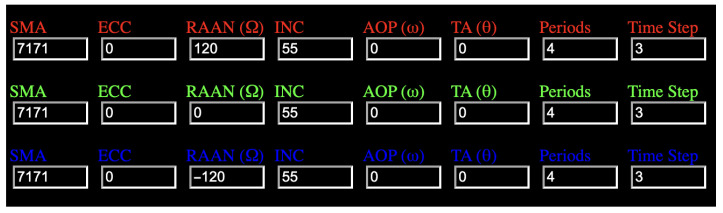
Orbital parameters of the designed LEO constellation.

The constellation considered in this study consists of six orbital planes with a total of 36 satellites. For clarity of illustration, only three representative orbital planes and one satellite in each plane are shown in the following figures, while the actual constellation contains denser satellite distributions. The basic orbital parameters used in the simulation are shown in Figure 2.

**Figure 3:**
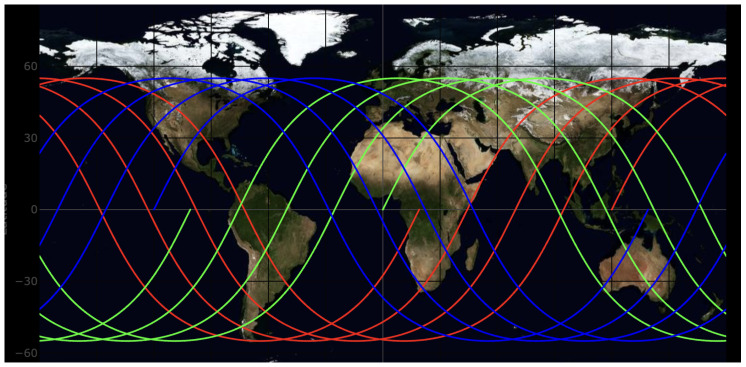
Subsatellite ground tracks of satellites from different orbital planes.

To illustrate the geometric relationship between satellites located in different orbital planes, the subsatellite ground tracks of satellites from several orbital planes are plotted in Figure 3. The figure shows the relative distribution of ground tracks for satellites in different planes within one day.

From the ground-track distribution, it can be observed that satellites in different orbital planes periodically approach and separate from each other as the Earth rotates. As a result, the inter-plane line-of-sight visibility also appears in clusters, similar to the satellite–ground visibility pattern summarized in Table 1.

Because the constellation consists of multiple orbital planes, the visibility conditions are complementary. When satellites in one orbital plane are not visible from the ground station, satellites in other planes may still have ground contact. In this case, the timing information synchronized with the ground station can be propagated to neighboring satellites through inter-satellite links.

#### 2.1.5. Star–Ground and Inter-Satellite Time Offset Measurement

**Figure 4:**
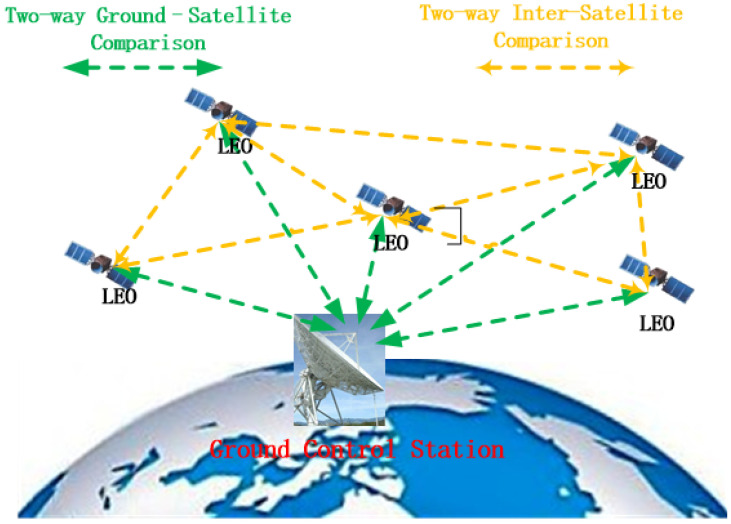
Conceptual diagram of star–ground and inter-satellite clock offset measurement.

During the visibility window, when the satellite can establish a link with the ground station or neighboring satellites as described in the preceding sections, star–ground or inter-satellite time comparison can be performed. Figure 4 illustrates the measurement concept. In the communication link, both ends perform signal acquisition, tracking, and data demodulation. Pseudorange and carrier-phase observables provide relative measurements such as range and range rate [24]. However, one-way observables generally embed the clock offset together with other error sources. Two-way time transfer mitigates these effects by enabling estimation of the true star–ground clock offset.

In a standard two-way time transfer model, the downlink and uplink observables can be written as

(2) $$\begin{array}{cc} P_{sg} & =\Delta T_{g}-\Delta T_{s}+\rho_{sg}+I_{sg}+T_{sg}\\ \; & \;+D_{g}^{T}+D_{s}^{R}+R_{sg}+\Delta \tau_{sg}+E_{s}^{T}+\varepsilon_{sg}, \end{array}$$

and

(3) $$\begin{array}{cc} P_{gs} & =\Delta T_{s}-\Delta T_{g}+\rho_{gs}+I_{gs}+T_{gs}\\ \; & \;+D_{s}^{T}+D_{g}^{R}+R_{gs}+\Delta \tau_{gs}+E_{s}^{R}+\varepsilon_{gs}, \end{array}$$

where $\Delta T_{g}$ and $\Delta T_{s}$ denote the ground and onboard clock offsets, and ρ, *I*, *T*, *D*, *R*, Δτ, and *E* represent the geometric, ionospheric, tropospheric, hardware-delay, relativistic/Sagnac, residual correction, and phase-center terms, respectively [25]. By differencing the two directions and dividing by 2, the estimated clock offset becomes

(4) $$\begin{array}{cc} \Delta T_{sg} & =\Delta T_{s}-\Delta T_{g}\\ \; & =\frac{\left(P_{gs}-P_{sg}\right)+\left(\rho_{sg}-\rho_{gs}\right)+\left(I_{sg}-I_{gs}\right)+\left(T_{sg}-T_{gs}\right)}{2}\\ \; & \;+\frac{\left(D_{g}^{T}-D_{s}^{T}\right)+\left(D_{s}^{R}-D_{g}^{R}\right)+\left(R_{sg}-R_{gs}\right)+\left(\Delta \tau_{sg}-\Delta \tau_{gs}\right)}{2}\\ \; & \;+\frac{\left(E_{s}^{T}-E_{s}^{R}\right)+\left(\varepsilon_{sg}-\varepsilon_{gs}\right)}{2}, \end{array}$$

which shows that orbit-related and geometry-related effects are not fully removed but enter through the residual term $(\rho_{sg}-\rho_{gs})/2$, thereby reducing—rather than eliminating—the sensitivity to OD errors compared with one-way transfer.

In practice, the achievable performance of two-way time transfer is strongly affected by calibration uncertainty of terminal/internal delays. State-of-the-art TWSTFT link calibration reports an uncertainty at the ∼1 ns level (mobile-station calibration) and about 1.5 ns (1σ) using a GPS calibrator [26]. This calibration uncertainty characterizes the $\sigma_{\mathrm{cal}}$ term, while the residual OD-/geometry-related contribution (e.g., $(\rho_{sg}-\rho_{gs})/2$) can be significantly larger in LEO scenarios and must be accounted for at the system level.

During non-visibility periods, star–ground time offset measurement is suspended and resumed when the satellite re-enters the visibility window. If inter-satellite links (ISLs) are available, clock offset measurement and forwarding can be used to bridge star–ground measurement gaps, particularly when ground station coverage is insufficient or when some satellites cannot communicate with the ground for extended periods [27]. In this study, an alternating “visible–non-visible” a+b pattern is used to emulate a worst-case measurement condition. With ISL supplementation, improved synchronization performance is expected [28,29].

### 2.2. Key Parameter Characterization

To support accurate control-oriented modeling and feasible controller design, key parameters were measured and evaluated. First, statistical analysis was conducted on time offset measurement data to quantify the measurement noise characteristics as a reference source. Second, long-term tests were performed on the adopted high-stability oscillator (OCXO) to characterize temperature sensitivity and aging trends. These parameters, including measurement noise, process noise, temperature-induced frequency drift, and aging drift, are essential for high-precision time synchronization and autonomous maintenance [30].

#### 2.2.1. Assessment of Star–Ground Time Offset Measurement Precision

At the current stage, a ground GNSS receiver 1PPS signal was used as an equivalent master clock input [31]. A time interval error (TIE) measurement device was employed to measure the time offset between the local system and the master clock, enabling ground-based validation of the control scheme.

To quantify the measurement noise floor, two identical high-precision GNSS timing receivers were used. Their 1PPS outputs were fed into the TIE measurement device for mutual comparison to obtain time difference data [32]. Due to environmental disturbances, the measured time difference may exhibit jumps [33]. The time series was preprocessed to remove outliers and abnormal segments, improving stability and validity. Specifically, MATLAB-2024b’s built-in isoutlier function was used for initial screening, followed by additional removal of abnormal intervals based on jump characteristics. The cleaned time series was then analyzed using metrics including RMS, extrema, and peak-to-peak values [34].

Assuming two identical devices with independent errors and equal variances, if the RMS of the measured difference is *R*, the RMS error σ of a single device is given by:

(5) $$\sigma =\frac{R}{\sqrt{2}}.$$

**Figure 5:**
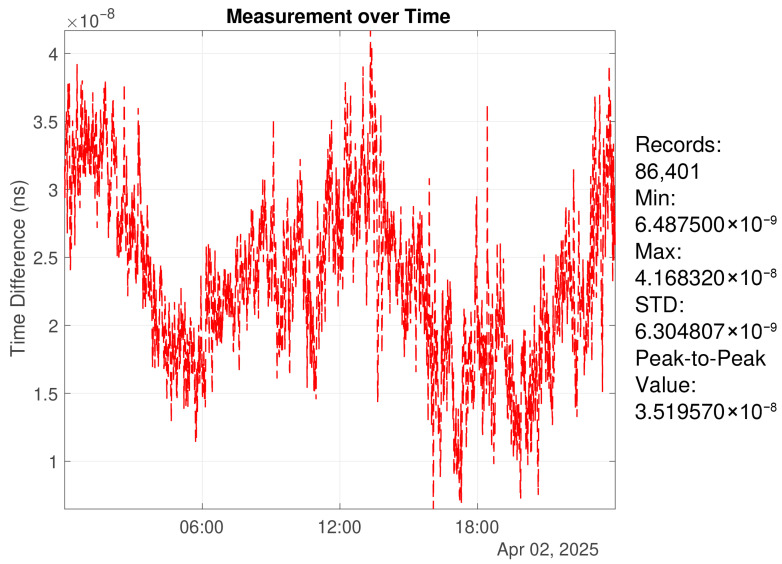
Mutual comparison results of 1PPS signals from two identical GNSS timing receivers.

Figure 5 shows a one-day mutual comparison result. The 1PPS RMS reaches the ∼2 ns level, indicating a low measurement noise floor and providing a reliable time reference for subsequent frequency disciplining experiments.

#### 2.2.2. Oscillator Specifications

In this work, both time synchronization and autonomous maintenance rely on a crystal oscillator as the frequency source. To satisfy the requirements for high precision and stability, frequency stability and phase noise performance are considered. Table 2 lists the major specifications of the selected domestic low-cost OCXO (ST36C010-12V).

#### 2.2.3. Development of the Voltage Control Circuit

To precisely tune the oscillator frequency, a voltage control board was developed based on an AD5791 high-resolution DAC, providing microvolt-level output resolution (theoretical LSB ≈ 1.955 μV). The board integrates low-noise reference sources (LT6654 for a −1.25 V reference and ADR4533 for a +3.3 V reference) and precision operational amplifiers. Power regulation stages (LT8608, LT3581, LT1963, LT3032, etc.) were used to ensure stable and low-noise supply rails for both the DAC and the oscillator.

**Figure 6:**
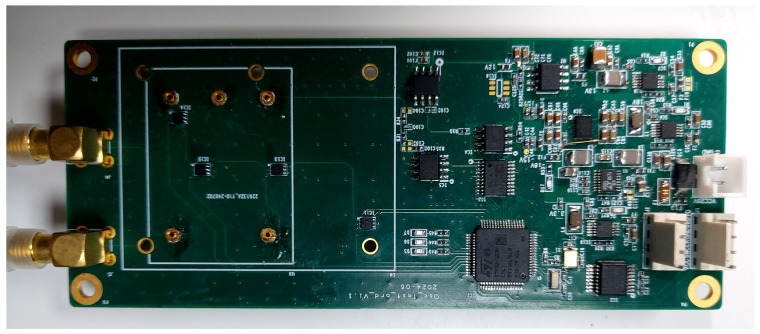
Developed OCXO voltage control board with integrated temperature sensors.

Four TMP117 high-precision digital temperature sensors (resolution 0.0078 °C, typical accuracy ±0.1 °C) were installed near the OCXO to monitor its operating temperature in real time. Temperature readings are transmitted to the MCU (STM32F103) via an I^2^C bus. The MCU uploads the control voltage and four temperature measurements to the host computer once per second via an RS-232 interface, and receives voltage commands at 1 Hz. The AD5791 output is driven via SPI to generate the required tuning voltage applied to the OCXO control input. The control board is illustrated in Figure 6.

Using extensive experimental data, a temperature–frequency mapping model α(T) can be established, providing a data basis for subsequent temperature feedforward compensation and enabling high-precision frequency control.

#### 2.2.4. Oscillator Parameter Measurements

##### Voltage-to-Frequency Sensitivity

The developed control board operates around a center tuning voltage of 2.5V and supports a tuning range from 1.25V to 3.3V. The minimum voltage step is approximately 2μV, enabling fine frequency adjustment. Because the voltage-to-frequency response varies across devices, the relationship between control voltage *V* and output frequency *f* must be identified. To mitigate temperature effects, the frequency response to voltage was measured at a stable temperature. Multiple datasets were collected, and the voltage sensitivity coefficient κ was extracted using least squares, serving as a quantitative basis for subsequent control modeling.

##### Piecewise Temperature Coefficient

In practice, OCXO frequency is affected by both tuning voltage and temperature. With voltage held constant, the frequency variation with temperature was measured [35]. Because the temperature–frequency relationship is nonlinear, a piecewise linear approximation was adopted after extensive measurements. The temperature coefficient α is defined in Hz/°C or, in normalized form, ppb/°C. The piecewise definition is given by:

(6) $$\alpha \left(T\right)=\left\{\begin{array}{cc} k_{1}, & \mathrm{if}\;a_{1}<T<a_{2}\\ k_{2}, & \mathrm{if}\;a_{2}<T<a_{3}\\ k_{3}, & \mathrm{if}\;a_{3}<T<a_{4}\\ \;\vdots \\ k_{n}, & \mathrm{if}\;T>a_{n} \end{array}\right.\;\mathrm{unit}:\;\mathrm{ppb}/{}^{\circ}C.$$

##### Evaluation of Aging Drift

To evaluate oscillator aging, the initial time offset $x_{0}$ was measured by comparing against a high-precision external reference (e.g., GNSS 1PPS) under a relatively stable temperature condition. The tuning voltage was adjusted such that the first-order frequency offset term $x_{1}$ converged to zero, thereby suppressing first-order drift contributions to time error accumulation. After compensating $x_{1}$, the residual time offset evolution is dominated by the second-order term $x_{2}$ (i.e., the frequency drift rate), which reflects the aging trend.

Repeated experiments were conducted over multiple days in a temperature-stable environment to monitor the evolution of time offset. With sufficiently long datasets covering typical aging behavior, a second-order polynomial fit was applied using least squares to estimate $x_{2}$. This approach separates aging effects while reducing the impact of incidental noise, providing a basis for model-based frequency prediction and phase compensation under GNSS-denied conditions.

In addition, once multi-day time offset data are available and a second-order fit is completed, the fitting residual can be used to estimate the process noise intensity of state evolution. Its variance can serve as a basis for the process noise covariance matrix *Q*, providing a statistically grounded prior for subsequent Kalman-filter-based dynamic estimation of aging-related states.

Detailed parameter estimation results for the oscillator, including the voltage-to-frequency sensitivity, piecewise temperature coefficient, and aging drift, are provided in Appendix A.

### 2.3. Time Synchronization and Autonomous Maintenance Method

**Figure 7:**
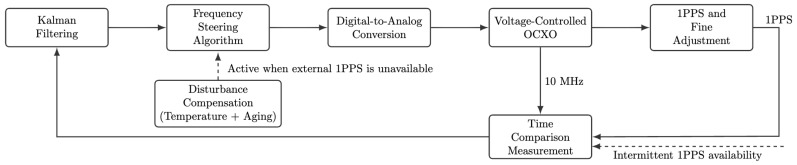
Workflow of time offset measurement, synchronization, and holdover in the LEO constellation scenario.

The workflow of remote synchronization and local maintenance for the LEO constellation is as follows. The system first measures the time offset between the local 1PPS signal and the remote reference 1PPS signal using a time-offset measurement module [36]. The measured time offsets are fed into a Kalman filter to obtain optimal estimates of time offset and frequency offset [37]. Based on the filter outputs, the frequency disciplining algorithm computes a control action that is converted into an analog voltage through a D/A module and applied to the OCXO, enabling real-time tuning of the output frequency. The tuned 10 MHz signal is used for 1PPS generation and fine adjustment, and is also fed back to the time-offset measurement module to form a closed-loop control structure, as illustrated in Figure 7.

When time-offset measurements are unavailable (corresponding to non-visibility intervals), the system enters a holdover state. In this mode, frequency is predictively compensated using calibrated oscillator parameters obtained during normal tracking, together with real-time temperature measurements, to maintain consistency between the local time and the remote time reference until measurements resume and closed-loop tracking is re-established.

Based on the oscillator model and the developed hardware platform, a time synchronization control algorithm combining closed-loop disciplining and open-loop holdover was designed. The algorithm supports smooth switching between the two modes, control action computation, and real-time prediction of oscillator states, ensuring high-precision synchronization across different operating conditions.

#### 2.3.1. Temperature-Augmented State-Space Modeling

**Figure 8:**
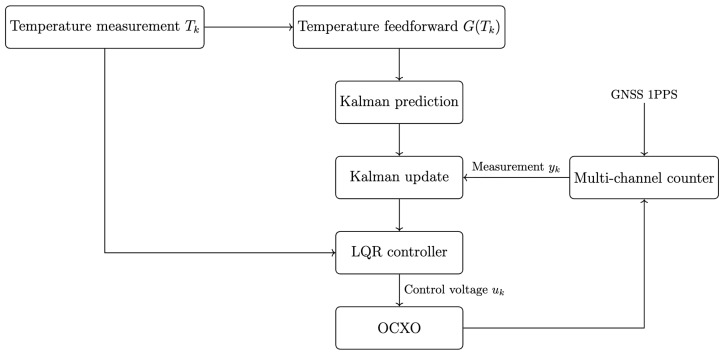
Control architecture with temperature feedforward compensation, Kalman filtering, and optimal control.

Using measured time offset observations together with the identified key parameters, a three-stage strategy of “temperature feedforward compensation + Kalman filter state estimation + optimal control” was adopted, as shown in Figure 8. To address the sensitivity of OCXO frequency to temperature disturbances and aging drift, a temperature–frequency mapping model is first established to remove temperature-induced variations online. Subsequently, a state-space model driven by time offset observations is formulated, and a Kalman filter is applied to jointly estimate frequency offset and drift rate.

##### State Definition

A commonly used representation for time offset dynamics is a second-order time model:

(7) $$T\left(t\right)=x_{0}+x_{1}t+\frac{1}{2}x_{2}t^{2}+\xi \left(t\right),$$

where T(t) denotes the time offset between the ideal reference time and the controlled clock at time *t* (unit: ns). This model represents the time offset as the combination of an initial bias, a frequency offset, and a frequency drift rate, while ξ(t) accounts for external disturbances and measurement errors.

The physical meanings of $x_{0}$, $x_{1}$, and $x_{2}$ are:

- $x_{0}$: time bias (time offset), unit: ns
- $x_{1}$: frequency offset, unit: ppb
- $x_{2}$: frequency drift rate (second-order drift/aging-related term), unit: ppb/s

The discrete-time state vector at epoch *k* is defined as:

(8) $$x_{k}=\left[\begin{array}{c} x_{0}\\ x_{1}\\ x_{2} \end{array}\right].$$

##### Temperature Feedforward Term

Temperature explicitly affects the frequency offset through the fitted function $\alpha (T_{k})$:

(9) $$G\left(T_{k}\right)=\left[\begin{array}{c} 0\\ \alpha (T_{k})\\ 0 \end{array}\right].$$

##### State Transition Model

The discrete-time state transition model is:

(10) $$x_{k+1}=\underset{A}{\underset{︸}{\left[\begin{array}{ccc} 1 & \tau & \frac{1}{2}\tau^{2}\\ 0 & 1 & \tau \\ 0 & 0 & 1 \end{array}\right]}}x_{k}+\underset{B}{\underset{︸}{\left[\begin{array}{c} 0\\ \kappa \\ 0 \end{array}\right]}}u_{k}+\underset{G(T_{k})}{\underset{︸}{\left[\begin{array}{c} 0\\ \alpha (T_{k})\\ 0 \end{array}\right]}}+w_{k},$$

where τ is the sampling interval (s), κ is the voltage-to-frequency sensitivity (Hz/V or ppb/V), and $w_{k}$ denotes the process noise. In this work, the variance of polynomial fitting residuals from the aging characterization experiments is used as a statistically grounded prior to construct the process noise covariance for Kalman filtering.

##### Measurement Model

Only the time bias $x_{0}$ is observed:

(11) $$y_{k}=\underset{H}{\underset{︸}{\left[\begin{array}{ccc} 1 & 0 & 0 \end{array}\right]}}x_{k}+v_{k},$$

where $v_{k}$ denotes measurement noise. The measurement noise covariance $R=\mathrm{Var}(y_{k})$ is determined from the receiver measurement precision evaluation.

##### Kalman Filtering Procedure (Pseudo-Code)

% Prediction

x_pred = A * x_prev + B * u_prev + G_of_T(T_k);

P_pred = A * P_prev * A’ + Q;

% Measurement update

y_pred = H * x_pred;

K = P_pred * H’ / (H * P_pred * H’ + R);

x_est = x_pred + K * (y_meas - y_pred);

P_est = (eye(3) - K * H) * P_pred;

#### 2.3.2. LQR-Based Optimal Control

##### Control Objective

The LQR controller computes an optimal control input (tuning voltage) based on the current state estimate to drive the oscillator system such that the states—in particular the accumulated time bias—converge to the desired values rapidly yet smoothly.

Given the state estimate:

(12) $${\hat{x}}_{k}=\left[\begin{array}{c} {\hat{x}}_{0}\\ {\hat{x}}_{1}\\ {\hat{x}}_{2} \end{array}\right],$$

the components represent the accumulated time bias, the instantaneous frequency offset relative to the reference (e.g., GNSS), and the drift trend, respectively.

##### Feedback Control Law

The LQR control law is:

(13) $$u_{k}=-K{\hat{x}}_{k},$$

or equivalently,

(14) $$u_{k}=-k_{0}{\hat{x}}_{0}-k_{1}{\hat{x}}_{1}-k_{2}{\hat{x}}_{2}.$$

The gain matrix *K* is obtained using MATLAB’s dlqr function:

(15) $$K=\mathrm{dlqr}(A,B,Q_{c},R_{c}).$$

The LQR formulation minimizes:

(16) $$J=\sum_{k=0}^{\infty }\left({\hat{x}}_{k}^{T}Q_{c}{\hat{x}}_{k}+u_{k}^{T}R_{c}u_{k}\right),$$

where $Q_{c}$ is the state weighting matrix reflecting performance priorities (e.g., time bias vs. frequency smoothness), and $R_{c}$ penalizes excessive control effort to avoid aggressive voltage variations.

The control output $u_{k}$ is a scalar voltage (V) applied to the OCXO control input via a DAC and the voltage control circuit to continuously fine-tune the oscillator frequency. By suppressing ${\hat{x}}_{1}$, the controller prevents further growth of the accumulated time bias ${\hat{x}}_{0}$ and drives it toward convergence. The inclusion of ${\hat{x}}_{2}$ enables anticipation of drift evolution and supports predictive compensation. The tuning of the weighting matrices $Q_{c}$ and $R_{c}$ determines the relative importance of time bias suppression, frequency stabilization, and control smoothness. In this work, $Q_{c}$ penalizes the phase error, frequency offset, and drift components of the state vector, while $R_{c}$ limits the magnitude of the control voltage applied to the OCXO. Increasing the phase weight accelerates the removal of accumulated time bias, while increasing the frequency weight suppresses short-term frequency fluctuations and improves output smoothness. Increasing the drift weight enhances compensation of slow variations such as aging and temperature effects. Therefore, by adjusting the relative weights of $Q_{c}$ and $R_{c}$, the controller achieves a practical balance between rapid time bias correction and smooth frequency regulation.

#### 2.3.3. Mode Switching Between Closed-Loop Locking and Open-Loop Prediction

The control system switches its operating mode based on the availability of the reference timing signal. When the reference is available and reliable, the system operates in a closed-loop disciplining mode. If the reference signal is lost or abnormal (e.g., consecutive missing 1PPS pulses or timing quality below a threshold), the system automatically transitions to an open-loop holdover mode.

Given the orbital period and the ground station visibility duration, an a+b minute synchronization cycle is employed, following the strategy of “*a* minutes of closed-loop synchronization + *b* minutes of open-loop holdover” repeatedly. To handle extreme situations such as severe weather or strong interference, the system records consecutive failures of closed-loop operation. For example, if one closed-loop window is missed, the system performs autonomous holdover for a duration of a+2b, and so forth, until closed-loop locking becomes available again.

To avoid abrupt frequency changes during mode switching, a phase-smoothing strategy is used. The open-loop prediction state is initialized based on the last closed-loop calibration result, allowing the time bias to decrease gradually over the prediction interval and avoiding large frequency perturbations. When returning to closed-loop operation, the control input is ramped to transition the system smoothly to the new steady state. These mechanisms ensure frequency continuity and phase continuity during mode transitions.

#### 2.3.4. Open-Loop State Prediction and Correction During Holdover

##### Temperature Compensation in Holdover

During non-visibility intervals, the local oscillator enters an open-loop holdover state, and its frequency is inevitably influenced by ambient temperature variations. To mitigate temperature-induced drift, a dynamic compensation strategy is adopted based on the previously established piecewise temperature coefficient model α(T) and real-time temperature monitoring.

Specifically, four-channel temperature readings are uploaded once per second by the control board. Median filtering is applied to reduce local transient disturbances. The filtered temperature determines the current interval $\left(a_{i},a_{i+1}\right)$, from which the corresponding linear coefficient $k_{i}$ is obtained. Because the tuning voltage is held constant when entering holdover, the observed temperature change $\Delta T=T_{\mathrm{now}}-T_{\mathrm{ref}}$ maps to an estimated frequency deviation:

(17) Δf=α(T)·ΔT,

where $\alpha \left(T\right)=k_{i}$ with unit ppb/°C, and Δf describes the frequency deviation relative to the reference temperature.

The frequency compensation Δf is mapped to a voltage adjustment using the previously measured voltage sensitivity coefficient κ (Hz/V or ppb/V):

(18) $$\Delta V=\frac{\Delta f}{\kappa }.$$

Finally, the host computer sends commands through SPI to adjust the AD5791 output to the required tuning voltage ΔV, driving the OCXO control input to counteract temperature-induced frequency changes. Owing to the microvolt-level control resolution (approximately 2μV) and high-precision temperature sensing, the proposed scheme enables fine-grained frequency trimming and effectively suppresses temperature disturbance. This method can be interpreted as an equivalent negative feedback mechanism under open-loop conditions, thereby improving holdover performance.

##### Aging Compensation in Holdover

In addition to temperature-induced drift, oscillator aging is a non-negligible error source during autonomous holdover. Aging typically manifests as a slow frequency drift over time with a relatively stable trend, which can be modeled as a continuous function over short to medium durations. To achieve high-precision time maintenance, an aging compensation mechanism is introduced in open-loop operation.

In the characterization stage, multi-day monitoring under stable temperature conditions was conducted with respect to a GNSS 1PPS reference. Least squares fitting was applied to the time offset series to extract the first-order frequency offset term $x_{1}$ and the second-order drift-rate term $x_{2}$, representing instantaneous frequency error and aging trend, respectively. Because $x_{2}$ is mainly driven by long-term physical changes of the oscillator and slow circuit variations, it is suitable for predictive compensation during holdover.

During holdover operation, a periodic correction (120 min in the current experiment) is adopted. Within each correction period, $x_{2}$ is updated using the most recent *N* periods (e.g., N=3–5) via a weighted moving average or an exponential recursive filter to produce an aging prediction term ${\hat{x}}_{2}$. The accumulated aging-induced frequency deviation over time *t* within the current period is predicted as:

(19) $$\Delta f_{\mathrm{aging}}\left(t\right)={\hat{x}}_{2}\cdot t,$$

which is then converted into a voltage compensation using κ:

(20) $$\Delta V_{\mathrm{aging}}=\frac{\Delta f_{\mathrm{aging}}}{\kappa }.$$

During second-by-second control, $\Delta V_{\mathrm{aging}}$ is added to the baseline tuning voltage, enabling dynamic compensation of aging drift. Given the small magnitude of $x_{2}$, the compensation can also be applied in a time-distributed manner across the *b*-minute holdover interval, achieving smoother frequency correction.

## 3. Results

### 3.1. Experimental Platform and Test Methodology

The experimental platform consists of a GNSS receiving antenna and timing module, a self-developed OCXO disciplining control board, high-precision time interval measurement equipment, host computer software, and data post-processing tools. During testing, the 1PPS signal generated by the GNSS receiver is used as the reference time source, while the 1PPS output of the locally controlled OCXO system is recorded simultaneously.

To emulate realistic constellation operation conditions, an a+b synchronization cycle is adopted, where a=7 min and b=113 min. Specifically, the 7 min interval corresponds to successful establishment of the star–ground timing link, during which time offset measurements are continuously available to evaluate closed-loop disciplining performance. The subsequent 113 min interval represents interruption of the star–ground link, during which the system enters open-loop holdover operation until the next synchronization opportunity, enabling assessment of autonomous time maintenance performance. The establishment and interruption of the timing link are implemented via scheduled software tasks.

**Figure 9:**
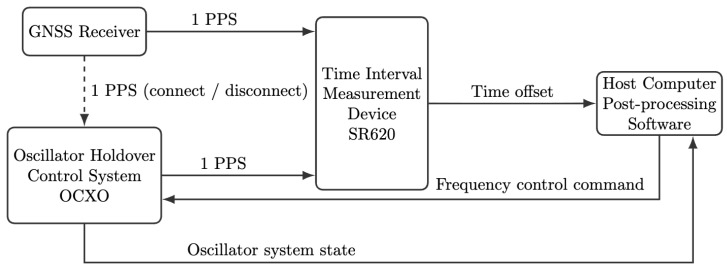
Physical connection diagram of the experimental platform.

Time offset measurements are performed using a high-precision time interval counter with a measurement accuracy better than 50 ps. The time difference between the local PPS and the reference PPS is recorded once per second and transmitted to the host computer for analysis. Based on these measurements, the host computer computes the required frequency control commands and sends them to the OCXO disciplining control system. A schematic of the experimental setup is shown in Figure 9.

### 3.2. Experimental Results and Analysis

#### 3.2.1. Free-Running Oscillator Performance

**Figure 10:**
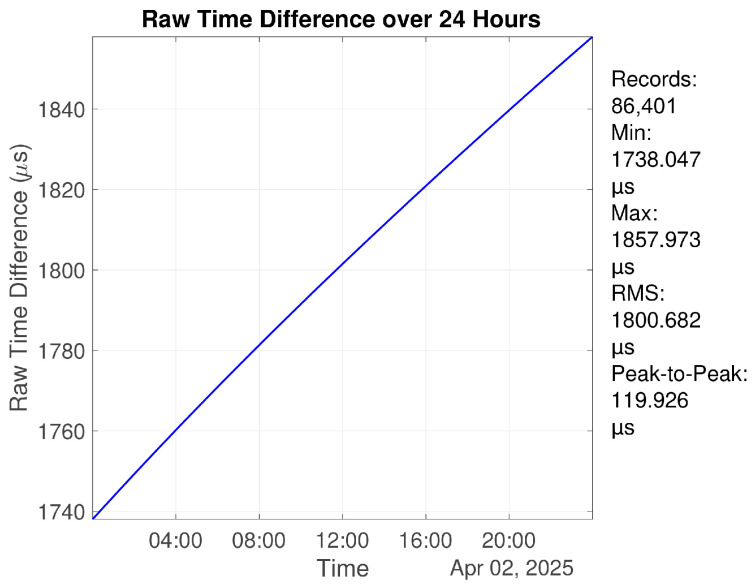
Time offset of the free-running OCXO over 24 h relative to the reference time.

**Figure 11:**
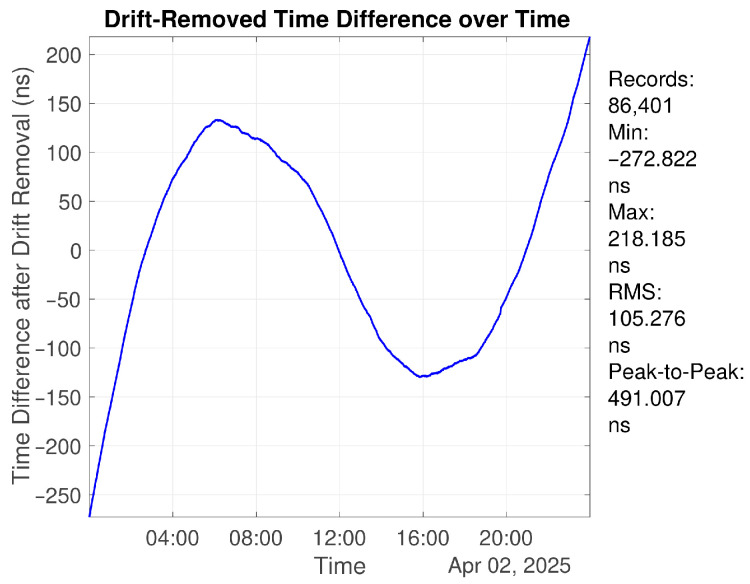
Residual time offset after removing first-order frequency drift over 24 h.

The OCXO system was first evaluated under free-running conditions at room temperature without applying the control algorithm. The time interval measurement device was used to measure the time offset between the OCXO-generated 1PPS and the GNSS 1PPS reference. Over a continuous 24 h period, the peak-to-peak time deviation reaches 119.926 μs. After removing the first-order frequency offset, the residual peak-to-peak time deviation is reduced to 491.007 ns over 24 h. The raw time offset and the residual after first-order drift removal are shown in Figure 10 and Figure 11, respectively.

#### 3.2.2. Performance Under Algorithm Control

To evaluate time synchronization accuracy and autonomous holdover performance under algorithmic control, experiments were conducted under the same conditions with the control algorithm enabled. The time interval measurement device was again used to measure the time offset between the OCXO-generated 1PPS and the GNSS reference.

**Figure 12:**
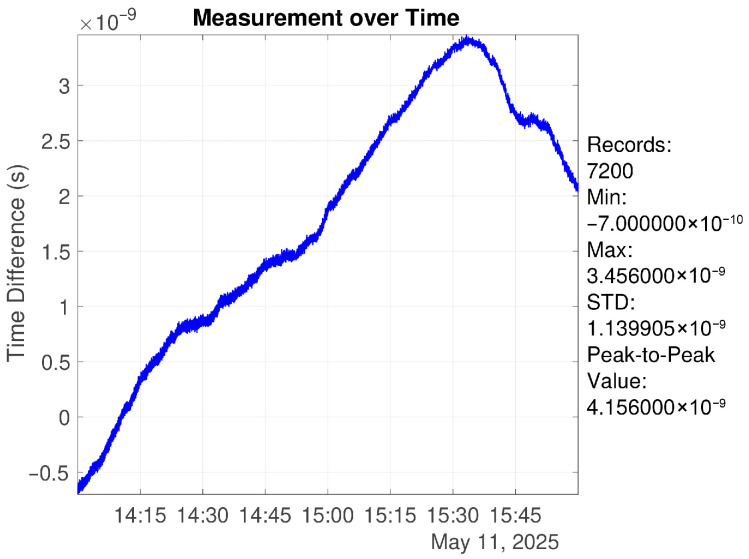
Time offset over 2 h under algorithm control (7 min closed-loop + 113 min open-loop).

**Figure 13:**
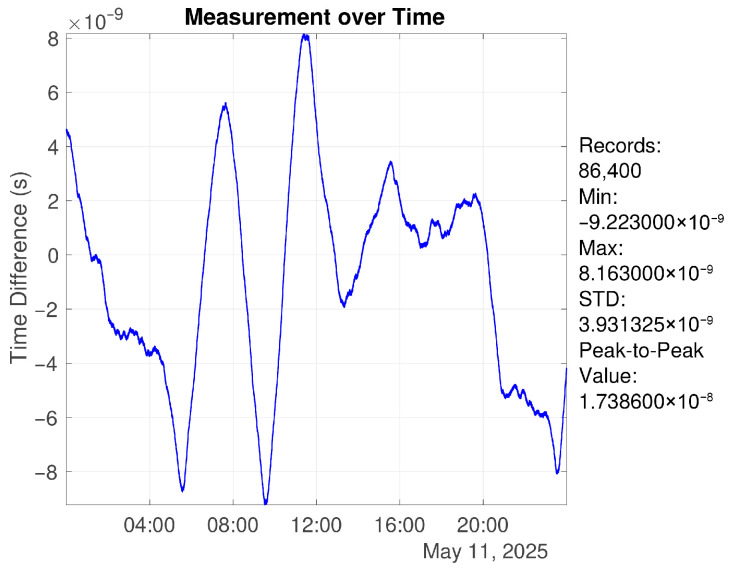
Time offset over 24 h under algorithm control (12 synchronization cycles).

Two scenarios were analyzed: a single synchronization cycle of 120 min (7 min closed-loop synchronization followed by 113 min open-loop holdover), and continuous operation over 24 h. The results show that over a 2 h period, the standard deviation of the time offset is 2.09 ns (1σ), with a peak-to-peak value of 4.156 ns, as shown in Figure 12. Over 24 h of continuous operation, the time offset standard deviation is 4.06 ns (1σ), with a peak-to-peak value of 17.386 ns, as shown in Figure 13.

Considering that the OCXO disciplining interval is set to 2 h (7200 s), the Allan deviation (ADEV) was evaluated at τ=10 s and τ=1000 s to characterize short-term and mid-to-long-term stability within a synchronization cycle. The results are summarized in Table 3. The short-term stability remains comparable between free-running and controlled conditions, indicating that the control algorithm does not degrade the intrinsic short-term stability of the oscillator. In contrast, long-term stability is significantly improved through temperature and aging compensation, enhancing time holdover capability by approximately one order of magnitude.

**Figure 14:**
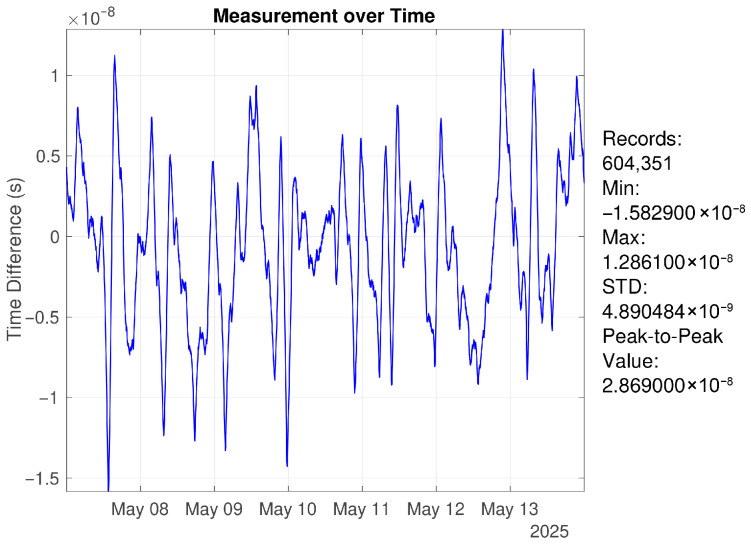
Timeoffset over one week of continuous operation, with peak-to-peak deviation better than 30 ns.

To further assess long-term performance, an additional continuous one-week experiment was conducted under identical conditions. The results indicate that over seven days of operation, the time offset standard deviation remains at 4.933 ns (1σ), with a peak-to-peak deviation of 28.69 ns, as shown in Figure 14.

Overall, the experimental results demonstrate that the proposed system can operate stably over long durations while maintaining an *method-induced* time error below 5 ns (1σ) under the assumed measurement model and synchronization schedule. The achieved synchronization performance meets the reference metrics discussed in the Introduction, and the method-induced 5 ns term does not constitute a dominant contribution in the overall timing error budget and satisfies the requirements corresponding to high resilience levels defined in emerging PNT standards. These results indicate strong engineering feasibility and potential applicability to time synchronization in LEO-PNT systems and other critical timing infrastructures.

## 4. Discussion

The experimental results demonstrate that the proposed time synchronization and autonomous holdover scheme can achieve nanosecond-level synchronization performance using a low-cost, high-stability crystal oscillator under intermittent star–ground synchronization conditions. In this section, the results are further interpreted in the context of existing time synchronization approaches, underlying mechanisms, and practical deployment considerations.

A key factor contributing to the achieved performance is the combined use of short-duration closed-loop synchronization and long-duration open-loop holdover. During the closed-loop phase, frequent time offset measurements enable rapid suppression of frequency bias through Kalman-filter-based state estimation and LQR-based optimal control. In the subsequent open-loop phase, temperature feedforward compensation and aging-aware prediction effectively mitigate the dominant error sources that typically limit crystal-oscillator-based timekeeping. This hybrid strategy allows the system to maintain high accuracy even when external timing references are unavailable for extended periods.

Compared with conventional approaches that rely heavily on continuous GNSS availability or onboard atomic clocks, the proposed method achieves a favorable balance between performance, cost, and system complexity. Atomic clocks offer superior long-term stability but incur significant penalties in terms of mass, power consumption, and cost, which limit their suitability for large-scale LEO constellation deployment. In contrast, the presented solution demonstrates that, when properly modeled and controlled, a low-cost OCXO can sustain time deviations well below the commonly referenced 50ns threshold, even over multi-day operation. This indicates that high-precision time maintenance is feasible for mission profiles with intermittent external anchoring and/or inter-satellite measurements, without universally eliminating the need for atomic clocks.

The Allan deviation analysis further confirms that the proposed control strategy preserves the intrinsic short-term stability of the oscillator while significantly improving mid- to long-term stability. This behavior is particularly important for constellation-based systems, where synchronization intervals are constrained by orbital geometry and ground station visibility. The improvement in long-term stability by approximately one order of magnitude highlights the effectiveness of incorporating temperature and aging compensation into the control framework.

From an application perspective, the ability of the system to directly output a local physical time signal without reliance on centralized post-processing or time prediction is a notable advantage. This feature supports decentralized and distributed time synchronization architectures, which are increasingly relevant for resilient positioning, navigation, and timing (PNT) services and for emerging LEO-based infrastructures. The demonstrated performance suggests that the proposed approach is compatible with stringent timing requirements in communication scheduling, beam management, and coordinated constellation operations.

Moreover, the compensation models adopted in this work are based on parametric fitting and linear prediction and have proven effective within the considered experimental time scales. For longer operation durations or more complex environmental conditions, data-driven techniques may serve as a complementary approach to further enhance long-term robustness.

Overall, the discussion indicates that the proposed method provides a practical and scalable solution for high-precision time synchronization and holdover in LEO constellations, under the assumed measurement model and operational conditions, bridging the gap between low-cost hardware and stringent system-level timing requirements.

## 5. Conclusions

This paper investigated autonomous time synchronization and holdover techniques for LEO satellite constellations under minimal external dependency. Based on orbital geometry and ground station visibility analysis, a periodic synchronization framework combining short-duration closed-loop calibration and long-duration open-loop holdover was established. A temperature-augmented oscillator drift model was formulated, and high-precision state estimation and optimal frequency control were realized through Kalman filtering and LQR-based regulation.

A complete hardware and software experimental platform was developed to validate the proposed approach using real physical measurements. Experimental results demonstrate that the system maintains method-induced time-error contributions at the level of 2.09 ns (1σ) over a 2 h synchronization cycle, 4.06 ns (1σ) over 24 h of continuous operation, and 4.93 ns (1σ) over one week, with peak-to-peak deviations below 30 ns. These results are consistent with the originally targeted 50 ns requirement when interpreted within a system-level error-budget context and confirm the effectiveness and robustness of the proposed method.

The proposed system achieves high-precision time synchronization using high-stability crystal oscillators together with intermittent short-duration star–ground time comparison links. This capability makes the system particularly suitable for decentralized time synchronization architectures in large-scale LEO constellations. The presented methodology provides a practical reference for the engineering implementation of high-precision timekeeping systems based on low-cost oscillators.

## Figures and Tables

**Table 1:** Typical daily visibility pattern of a single satellite.

Parameter	Value	Description
Orbital period	∼101 min	Orbit duration at 800 km altitude
Orbits per day	∼14	Total orbital revolutions per day
Consecutive visible passes	∼5	Continuous satellite–ground contacts
Non-visible orbital cycles	∼8	Periods without ground visibility
Ground-track repeat period	∼23.5 h	Approximate repetition of subsatellite track

**Table 2:** Specifications of the OCXO (ST36C010-12V).

Item	Specification
Nominal frequency	10.000 MHz
Output waveform	Sine wave (50 Ω load)
Frequency accuracy	≤±0.1 ppm (at +25 °C, EFC = 2.5±0.2 V)
Frequency stability @1 s	<$5\times 10^{-12}$
Phase noise @1 Hz	−100 dBc/Hz (warm-up >2 h)
Phase noise @10 Hz	−130 dBc/Hz
Phase noise @100 Hz	−150 dBc/Hz
Phase noise @1 kHz	−155 dBc/Hz
Phase noise @10 kHz	−158 dBc/Hz
Supply voltage	+12 VDC ($V_{\mathrm{cc}}\pm 5\%$, ripple <500 mV)
Tuning voltage range	0∼5 V (center +2.5 V)
Tuning range	≥±0.8 ppm
Signal power	9±2 dBm (50 Ω load)
Harmonic suppression	<−40 dBc
Spurious suppression	<−80 dBc

**Table 3:** Comparison of Allan deviation (ADEV) under different operating conditions.

Averaging Time	Free-Running	Disciplined OCXO
ADEV at 10 s	$4.668\times 10^{-12}$	$7.718\times 10^{-12}$
ADEV at 1000 s	$3.193\times 10^{-12}$	$6.544\times 10^{-13}$

**Figure A1:**
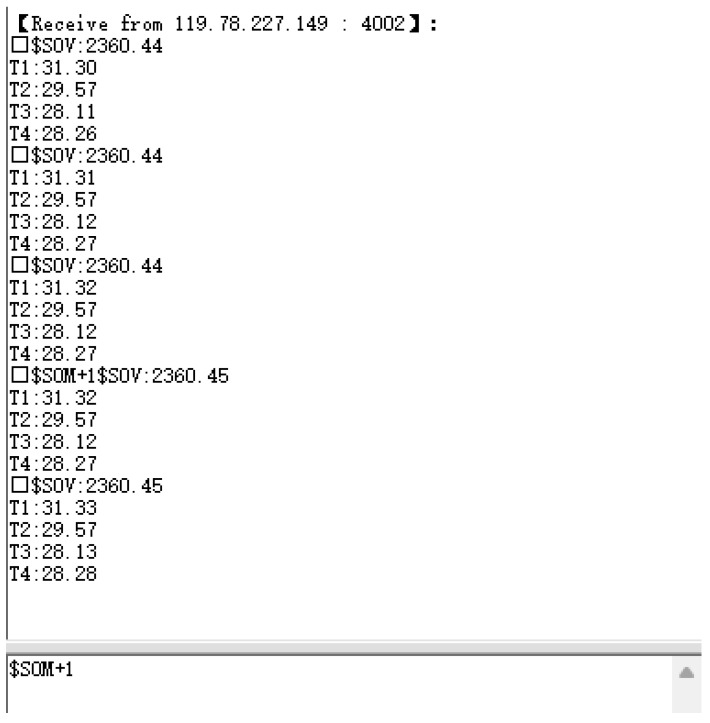
Voltage control command and monitoring interface showing DAC voltage adjustment.

**Figure A2:**
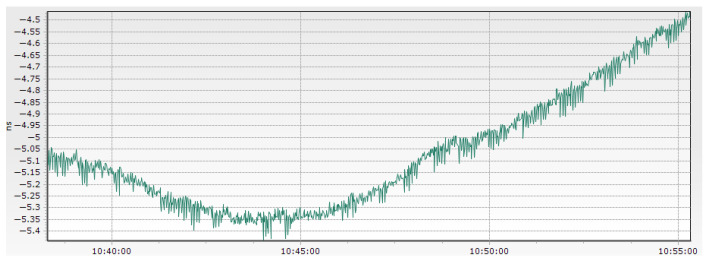
Measured time interval error (TIE) variation after applying a voltage adjustment.

**Figure A3:**
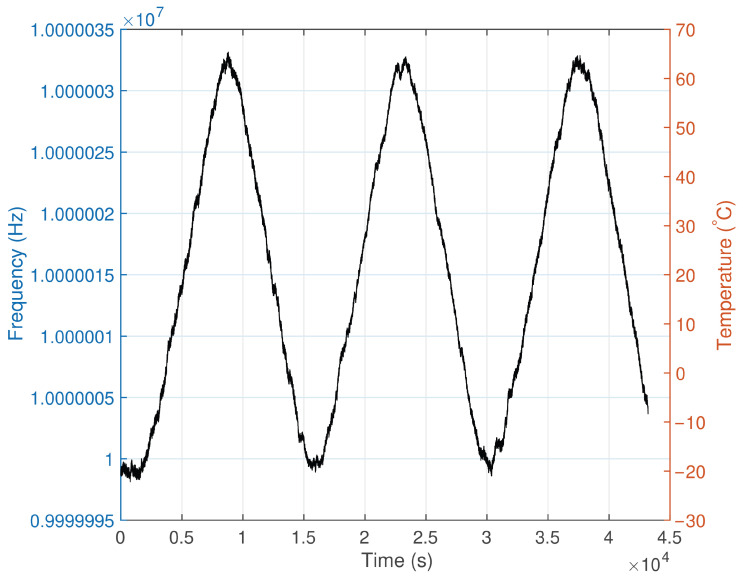
Measured relationship between temperature cycling and oscillator frequency variation.

## Data Availability

The data underlying this study is available from the corresponding author upon request.

## Abbreviations

The following abbreviations are used in this manuscript:

LEO	Low Earth Orbit
GNSS	Global Navigation Satellite System
OCXO	Oven-Controlled Crystal Oscillator
PPS	Pulse Per Second
TIE	Time Interval Error
ADEV	Allan Deviation
LQR	Linear Quadratic Regulator
PNT	Positioning, Navigation, and Timing
STL	Satellite Time and Location
DAC	Digital-to-Analog Converter

## Appendix A. Oscillator Calibration Data and Parameter Estimation

In this appendix, we present the experimentally derived calibration data of the OCXO, including the voltage-to-frequency sensitivity coefficient κ, the temperature-dependent frequency coefficient α(T), and the aging drift. These parameters were obtained from dedicated measurements of the oscillator under controlled operating conditions.

### Appendix A.1. Voltage-to-Frequency Sensitivity Measurement

The voltage-to-frequency sensitivity of the OCXO was measured in a temperature-stabilized environment at 23.5 °C. The 1 PPS signal generated by the oscillator was compared with the reference signal from a hydrogen maser. Voltage offsets were applied to the OCXO through RS232 commands to the DAC, and the resulting phase variation was monitored using a Time Interval Error (TIE) measurement device.

Figure A1 shows the serial monitoring interface, where the voltage control commands sent to the DAC can be observed. Figure A2 illustrates the corresponding phase variation measured by the TIE device after the voltage adjustment, demonstrating that the oscillator frequency responds to the applied control voltage.

By applying multiple voltage adjustments and recording the corresponding phase evolution, the frequency response of the oscillator to the control voltage was estimated. A least-squares fitting method was then used to determine the voltage-to-frequency sensitivity coefficient.

The experimental results show that a voltage change of 1.955μV corresponds to a fractional frequency offset of $0.776\times 10^{-13}$ for the tested OCXO.

### Appendix A.2. Aging Drift Estimation

The aging behavior of the OCXO was evaluated through long-term measurements conducted under a constant temperature environment. After compensating the nominal frequency offset, the time difference between the OCXO 1 PPS signal and the hydrogen maser reference was continuously recorded using the TIE measurement system for approximately one week.

For precision crystal oscillators, the long-term aging behavior after the initial burn-in stage can generally be approximated as a linear frequency drift. Therefore, the oscillator frequency variation with time can be expressed as

(A1) f(t)=a+bt

where *a* represents the initial frequency offset and *b* denotes the aging rate of the oscillator.

Given a sequence of frequency measurements $f_{i}$ obtained at time instants $t_{i}$, the aging rate *b* can be estimated using the least-squares method

(A2) $$b=\frac{n\sum_{i=1}^{m}\left(f_{i}-\bar{f}\right)\left(t_{i}-\bar{t}\right)}{f_{0}\sum_{i=1}^{m}{\left(t_{i}-\bar{t}\right)}^{2}}$$

where $\bar{f}$ is the mean frequency of the *m* measurements, $\bar{t}$ is the mean observation time, and $f_{0}$ is the nominal oscillator frequency.

Based on the experimental dataset collected over approximately one week, the aging rate of the tested OCXO was estimated to be

0.232ppb/day.

After removing the linear aging trend, the remaining residual sequence mainly reflects stochastic fluctuations of the oscillator, which can be used to estimate the process noise in the oscillator frequency model used for frequency prediction and phase compensation.

### Appendix A.3. Temperature-to-Frequency Sensitivity

The temperature sensitivity of the OCXO was characterized over the temperature range from −25 °C to 60 °C. During the experiment, the oscillator was subjected to repeated temperature cycles with a period of approximately three hours, covering the range from about −20 °C to 60 °C. The corresponding frequency variations were measured simultaneously.

As shown in Figure A3, the oscillator frequency exhibits a clear dependence on temperature during the thermal cycling process.

To describe the temperature dependence of the oscillator frequency in a simple and practical manner, a piecewise linear model was adopted. The temperature range was divided into several intervals of approximately 20 °C, and the temperature coefficient within each interval was estimated using least-squares fitting.

The resulting temperature sensitivity function can be expressed as

(A3) $$\alpha \left(T\right)=\left\{\begin{array}{cc} 0.37\;\mathrm{Hz}/{}^{\circ}C, & -25\leq T<-5\\ 0.40\;\mathrm{Hz}/{}^{\circ}C, & -5\leq T<15\\ 0.43\;\mathrm{Hz}/{}^{\circ}C, & 15\leq T<35\\ 0.39\;\mathrm{Hz}/{}^{\circ}C, & 35\leq T\leq 60 \end{array}\right.$$

This piecewise representation provides an approximate description of the temperature-dependent frequency behavior of the oscillator within the measured operating range.
